# Supplementary material for: Photoswitchable Molecular Units with Tunable Nonlinear Optical Activity: A Theoretical Investigation
Source: Molecules. 2023 Jul 26;28(15):5646. doi: 10.3390/molecules28155646 (PMC10419997; doi:10.3390/molecules28155646)
Supplement: Supplementary file 1 [file molecules-28-05646-s001.zip › molecules-2515041-supplementary.pdf]

Photoswitchable Molecular Units with tunable Non-Linear Optical Activity: A Theoretical Investigation

Aggelos Avramopoulos, Heribert Reis, Demeter Tzeli, Robert Zalesny, Manthos, G . Papadopoulos

Supporting Information

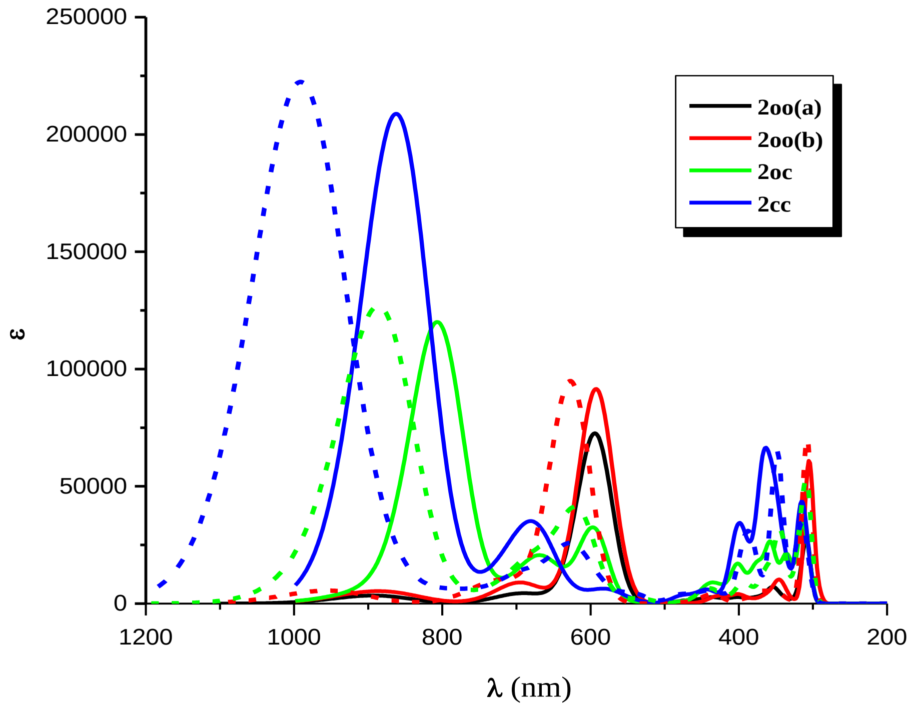

Figure S1. Absorption spectra of the **2oo**, **2oc**, and **2cc** structures at B3LYP(dotted line) and CAM-B3LYP(solid) /6-31G*H,C,O,F,S ECP28MWB(SDD)Ni level of theory. (Peak half-width at half height: 0.09 eV)

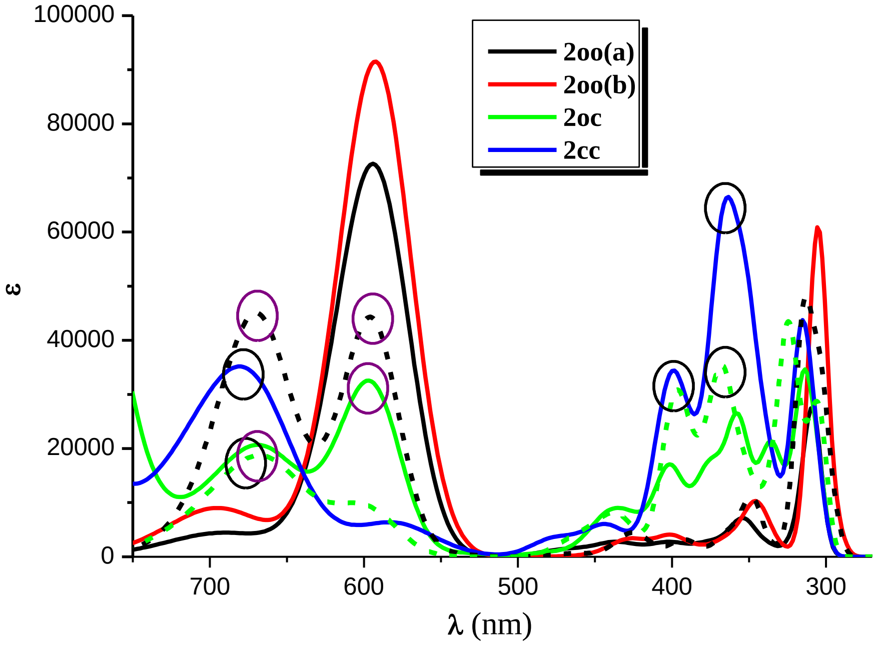

Figure S2 Vis-UV absorption (solid line) and emission (dotted line) spectra of the **2oo**, **2oc**, and **2cc** structures at CAM-B3LYP/6-31G*H,C,O,F,S ECP28MWB(SDD)Ni level of theory. (Peak half-width at half height: 0.09 eV)

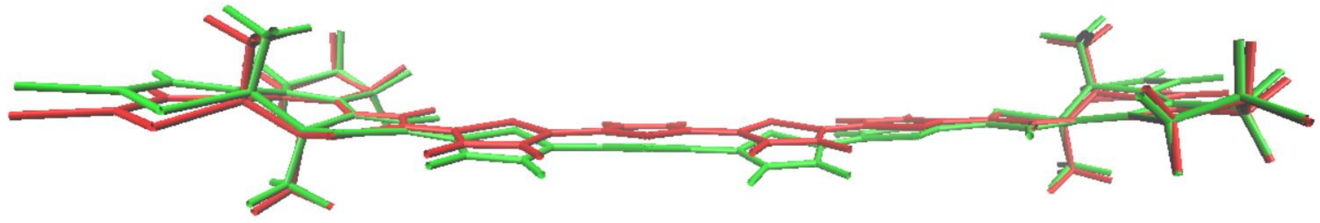

(**a**)

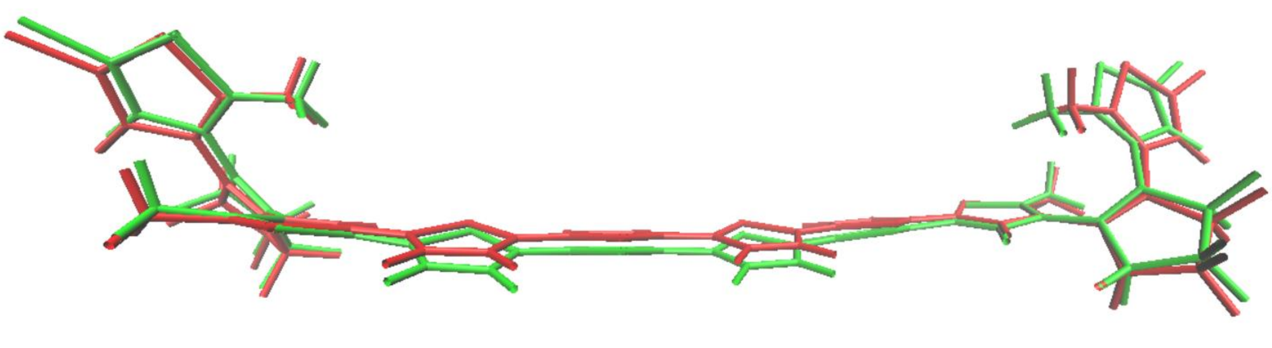

(**b**)

Figure S3. Comparison of the two optimized structures for **1cc(a)** and **1oo(b)** molecules, R=Cl (Figure 1). Red color depicts B3LYP/6-31G* optimized geometry, green color depicts the corresponding M062-X/6-31G*.

TABLE S1. The total energy (E;a.u.) HOMO(a.u), LUMO(a.u.), and the Homo-Lumo gap (Δ_{HL} =|H-L|;a.u.) of 1oo/oc/cc, 2oo/2oc/cc, and 3oo/cc derivatives (Figures 1&2).

Derivative	E[1]	HOMO[1]	LUMO[1]	Δ_{HL}[1]
**1oo**				
**R=H**	-6152.0066	-0.183	-0.085	0.098
**R=Cl**	-7071.1774	-0.186	-0.087	0.099
**R=NO_2**	-6590.9881	-0.192	-0.104	0.088
**R=NH_2**	-6262.7009	-0.181	-0.082	0.099
**R=NO_2/NH_2**	-6411.8448	-0.186	-0.102	0.084
**1oc**				
**R=H**	-6151.9873	-0.183	-0.105	0.078
**R=Cl**	-7071.1612	-0.187	-0.109	0.078
**R=NO_2**	-6590.9685	-0.198	-0.130	0.068
**R=NH_2**	-6262.6934	-0.172	-0.094	0.078
**R=NO_2/NH_2**	-6411.8367	-0.174	-0.103	0.071
**1cc**				
**R=H**	-6151.9678	-0.185	-0.111	0.074
**R=Cl**	-7071.1446	-0.189	-0.115	0.074
**R=NO_2**	-6590.9481	-0.202	-0.137	0.065
**R=NH_2**	-6262.6854	-0.171	-0.099	0.072
**R=NO_2/NH_2**	-6411.8174	-0.179	-0.128	0.051
**2oo**	-9230.0634	-0.226	-0.167	0.059
**2oc**	-9230.0781	-0.217	-0.167	0.050
		-0.215[2]	-0.179[2]	0.036[2]
**2cc**	-9230.0957	-0.210	-0.168	0.042
		-0.209[2]	-0.179[2]	0.030[2]
**3oo**	-7967.3531	-0.208	-0.157	0.051
**3cc**	-7697.3710	-0.210	-0.156	0.054

[1] (U)B3LYP/6-31G*

[2] (U) HSEH1PBE/6-31G*

Table S2. The magnitude of the dipole moment, μ, of 1cc, 1oc and 1oo (Figure 1). The structures have been optimized with the B3LYP/6-31G*. All values were computed with CAM-B3LYP/6-31G* method and given in a.u

.	μ		
R	**1cc**	**1co**	**1oo**
**H**	0.082 (0.091)[1]	1.213	1.650
**Cl**	0.390 (0.430)[1]	1.037	0.968
**NO_2**	0.179	1.974	0.543
**NH_2**	0.141	1.613	2.172
**Ph**	0.215	0.858	1.764
**NO_2/NH_2[2]** **NO_2/NH_2[3]**	5.162	4.808	2.861

[1] Basis set: cc-pVTZ (C,S,,H,Cl,F), SDD(Ni)

[2] NO_2/NH_2 groups are anchored on open/closed unit.

[3] NO_2/NH_2 groups are anchored on closed/open unit.

TABLE S3. The magnitude of the dipole moment, μ, of 2oo, 2oc, 2cc, 3oo and 3oc (Figure 2). The structures have been optimized with the B3LYP/6-31G*. All values were computed with (U)CAM-B3LYP/6-31G* method and given in a.u

**Derivative**	**μ**
2oo	1.646
2oc	2.054 (2.546)[1]
2cc	2.012 (2.578)[1]
3oo	1,739
3cc	1.689

[1] Basis set: cc-pVTZ (C,S,H,F), SDD(Ni)

Table S4: Excitation energy (ΔE, [eV]), oscillator strength (f) and two-photon transition strength (⟨δ^{2PA}⟩, [au]) corresponding to five lowest-energy electronic transitions for molecule R=Cl (Figure 1)

	**1cc**			**1oc**			**1oo**		
	**ΔE**	**f**	**<δ^{2PA}> ×10^3**	**ΔE**	**f**	**<δ^{2PA}> ×10^3**	**ΔE**	**f**	**<δ^{2PA}> ×10^3**
$S_0 \rightarrow S_1$	2.162	2.3537	<0.1	2.243	1.4665	7.0	2.820	2.5150	<0.1
$S_0 \rightarrow S_2$	2.344	0.0159	0.2	2.843	1.2518	113.3	3.543	0.0226	8.6
$S_0 \rightarrow S_3$	2.885	0.6755	0.4	3.436	0.0660	1512.2	3.994	0.0236	<0.1
$S_0 \rightarrow S_4$	3.340	0.0008	6690.7	3.648	0.0164	2358.2	4.023	0.0380	126.0
$S_0 \rightarrow S_5$	3.577	0.0002	3105.5	3.793	0.0868	19.5	4.156	0.0030	2023.3

Table S5: Excitation energy (ΔE, [eV]), oscillator strength (f) and two-photon transition strength (⟨δ^{2PA}⟩, [au]) corresponding to five lowest-energy electronic transitions for molecule R=H (Figure 1).

	**1cc**			**1oc**			**1oo**		
	ΔE	f	<δ^{2PA}> ×10^3	ΔE	f	<δ^{2PA}> ×10^3	ΔE	f	<δ^{2PA}> ×10^3
$S_0 \rightarrow S_1$	2.169	2.1698	<0.1	2.252	1.3319	3.2	2.819	2.4780	<0.1
$S_0 \rightarrow S_2$	2.344	0.0114	1.8	2.844	1.3222	94.1	3.544	0.0208	5.2
$S_0 \rightarrow S_3$	2.878	0.7472	0.3	3.450	0.0534	1192.0	4.045	0.0252	<0.1
$S_0 \rightarrow S_4$	3.353	0.0002	5802.2	3.643	0.0144	2168.1	4.080	0.0337	431.2
$S_0 \rightarrow S_5$	3.556	0.0002	2516.0	3.748	0.0822	25.3	4.162	0.0106	1657.9

Table S6: Excitation energy (ΔE, [eV]), oscillator strength (f) and two-photon transition strength (⟨δ^{2PA}⟩, [au]) corresponding to five lowest-energy electronic transitions for molecule R=NH_2 (Figure 1)

	1cc			1oc			1oo		
	ΔE	f	$<\delta^{2PA}>$ $\times 10^3$	ΔE	f	$<\delta^{2PA}>$ $\times 10^3$	ΔE	f	$<\delta^{2PA}>$ $\times 10^3$
$S_0 \rightarrow S_1$	2.161	2.4581	<0.1	2.253	1.5232	2.8	2.817	2.5106	<0.1
$S_0 \rightarrow S_2$	2.353	0.0150	31.9	2.885	1.2299	41.8	3.540	0.0226	2.6
$S_0 \rightarrow S_3$	2.935	0.5549	0.3	3.526	0.0218	1966.6	3.940	0.0263	1.0
$S_0 \rightarrow S_4$	3.382	0.0005	9561.7	3.562	0.0402	1807.8	3.949	0.0974	13.5
$S_0 \rightarrow S_5$	3.544	0.0002	1.6	3.905	0.0294	25.9	4.128	0.0018	1927.5

Table S7: Excitation energy (ΔE, [eV]), oscillator strength (f) and two-photon transition strength ($\langle\delta^{2PA}\rangle$, [au]) corresponding to five lowest-energy electronic transitions for molecule R=NO_2 (Figure 1)

	1cc			1oc			1oo		
	ΔE	f	$<\delta^{2PA}>$ $\times 10^3$	ΔE	f	$<\delta^{2PA}>$ $\times 10^3$	ΔE	f	$<\delta^{2PA}>$ $\times 10^3$
$S_0 \rightarrow S_1$	1.932	2.0938	<0.1	1.984	1.1765	49.1	2.820	2.5449	<0.1
$S_0 \rightarrow S_2$	2.068	0.0024	39.1	2.691	1.4493	367.0	3.539	0.0248	20.3
$S_0 \rightarrow S_3$	2.696	1.0551	<0.1	3.229	0.1597	3058.4	3.840	0.0130	0.4
$S_0 \rightarrow S_4$	3.089	0.0009	10167.0	3.374	0.1308	715.1	3.853	0.0056	20.2
$S_0 \rightarrow S_5$	3.322	0.0006	4760.5	3.468	0.0753	9.2	3.912	0.0003	<0.1

Table S8: Excitation energy (ΔE, [eV]), oscillator strength (f) and two-photon transition strength ($\langle\delta^{2PA}\rangle$, [au]) corresponding to five lowest-energy electronic transitions for molecule R=NO_2, R'=NH_2 (Figure 1)

	1cc			1oc[a)]			1oo		
	ΔE	f	$<\delta^{2PA}>$ $\times 10^3$	ΔE	f	$<\delta^{2PA}>$ $\times 10^3$	ΔE	f	$<\delta^{2PA}>$ $\times 10^3$
$S_0 \rightarrow S_1$	1.951	1.7422	118.4	2.249	1.5157	8.3	2.817	2.5218	3.5

$S_0 \rightarrow S_2$	2.243	0.6864	85.5	2.886	1.2587	9.6	3.538	0.0245	9.3
$S_0 \rightarrow S_3$	2.704	0.3358	1286.2	3.528	0.0035	3251.3	3.799	0.0068	9.3
$S_0 \rightarrow S_4$	3.002	0.3582	2916.7	3.542	0.0658	557.8	3.912	0.0002	0.2
$S_0 \rightarrow S_5$	3.253	0.0042	1586.6	3.777	0.0015	0.6	3.951	0.0583	5.3

[a] NO_2 group attached to "open" moiety.

Table S9: Excitation energy (ΔE, [eV]), oscillator strength (f) and two-photon transition strength (⟨δ^{2PA}⟩, [au]) corresponding to five lowest-energy electronic transitions for molecule R=NO_2, R'=NH_2. (Figure 1)

	**1oc**[a]		
	ΔE	f	$<\delta^{2PA}>$ $\times 10^3$
$S_0 \rightarrow S_1$	1.972	1.2258	70.5
$S_0 \rightarrow S_2$	2.650	1.3286	541.5
$S_0 \rightarrow S_3$	3.195	0.2143	3036.5
$S_0 \rightarrow S_4$	3.360	0.1164	743.6
$S_0 \rightarrow S_5$	3.460	0.0732	11.5

[a] NO_2 group attached to "closed" moiety.

TABLE S10. Electronic transition energies(E;eV) and oscillator strengths (f).

Dimer	D_n[a]	E/f[b]	Transition/(%)	A_n[a]	E/f[b]	Transition/(%)
**1oc**[c]	D_1	**4.433/0.139**	**HOMO->LUMO/(100)**	**A_7**	**4.233/0.04**	**HOMO-2->LUMO/21**
		**4.450/0.111**[d]			**4.320/0.01**[d]	**HOMO-1->LUMO+4/19**
						**HOMO->LUMO+4/27**
**2oc**	**D_{22}**	**4,619/0,22**	HOMO-14->LUMO (50%)	**A_{55}**	**4,607/0,04**	HOMO->LUMO+9(22%)
			HOMO->LUMO+2 (12.5%)			HOMO-9->LUMO+4 (11%)
			HOMO-5->LUMO+1 (14.5%)			HOMO-7->LUMO+3(5,8%)
						HOMO-1->LUMO+5(8%)
**4oc**	**D_1**	**4.226/0.702**	**HOMO->LUMO/(98)**	**A_4**	**4.176/0.124**	HOMO->LUMO+1/67
				**A_1**	2.628/0.458	HOMO->LUMO/95
				**A_2**	3.892/0.121	HOMO-1->LUMO/54
						HOMO-2->LUMO+1/32
**5oc**	**D_1**	**4.217/0.181**	**HOMO->LUMO/(98)**	**A_4**	**4.209/0.356**	HOMO->LUMO+1/63
				A_1	2.607/0.470	HOMO->LUMO/100
				A_2	3.908/0.108	HOMO-2->LUMO/84
				A_3	3.921/0.136	HOMO-1->LUMO/77
**6oc**	**D_1**	**4.217/0.702**	**HOMO->LUMO/(98)**	**A_4**	**4.210/0.356**	HOMO->LUMO+1/63
				A_1	2.607/0.689	HOMO->LUMO/100
				A_2	3.909/0.108	HOMO-2->LUMO/84
				A_3	3.921/0.136	HOMO-1->LUMO/77
**7oc**	D_1	2.482/0		A_6	1.345/0.37	HOMO->LUMO/92
	D_2	3.147/0		A_{24}	2.650/0.28	HOMO->LUMO+1/84
	D_3	3.627/0		A_{42}	3.770/0.15	HOMO-1->LUMO+1/79
	**D_4**	**4.231/0.174**	**HOMO->LUMO/(100)**	A_{46}	4.040/0.06	HOMO-2->LUMO+1/84
				**A_{50}**	**4.160/0.06**	**HOMO-6->LUMO/29**
						**HOMO-14->LUMO/25**
						**HOMO-3->LUMO/8**

**8oc**	D_1	2.456/0		A_6	1.383/0.51	HOMO->LUMO/92
	D_2	3.140/0		A_7	2.607/0.02	HOMO-1->LUMO/79
	D_3	3.620/0		A_{24}	2.600/0.215	HOMO->LUMO+1/87
	**D_4**	**4.205/0.173**	**HOMO->LUMO/(100)**	A_{32}	3.109/0.04	HOMO-2->LUMO/44
				A_{40}	3.730/0.141	HOMO-1->LUMO+1/79
				A_{47}	4.095/0.06	
				**A_{50}**	**4.136/0.01**	**HOMO-2->LUMO+1/40**<br>**HOMO->LUMO+2/37**

[a] D_n/A_n: n denotes the number of Donor/Acceptor state for the transition S_0->S_n

[b] E/f: Electronic Transition Energy(eV)/Oscillator Strength.

[c] R=Cl (Figure 1)

[d] Properties have been computed at the M062-X optimized geometry.

Table S11 Excitation energies, ΔE (eV), λ_{max} (nm), and f-values for the main peak of the absorption and emission spectra and the corresponding main excitations of the **2oo**, **2oc**, and **2cc** structures at B3LYP **[I]** and CAM-B3LYP **[II]**/6-31G*$_{H,C,O,F,S}$ ECP28MWB(SDD)$_{Ni}$ level of theory.

<table>
<tr><th>Struct</th><th>DFT</th><th>ΔE</th><th>λ_{max}</th><th>f</th><th>Excitations</th></tr>
<tr><td colspan="6">Absorption</td></tr>
<tr><td>2oo(a)</td><td>II</td><td>1.805</td><td>686.9</td><td>0.0115</td><td>0.35|H-2→L+1> – 0.29|H→L+2> + 0.28|H-1→L+1>
+ 0.26|H-2→L></td></tr>
<tr><td></td><td></td><td>2.071</td><td>598.7</td><td>0.1141</td><td>0.20|H→L+2> + 0.16|H-1→L+2> – 0.21|H-10→L+5></td></tr>
<tr><td></td><td></td><td>4.006</td><td>309.5</td><td>0.1252</td><td>0.19|H-6→L+2> – 0.13|H-1→L+6></td></tr>
<tr><td>2oo(b)</td><td>II</td><td>1.791</td><td>692.2</td><td>0.0447</td><td>0.56|H-1→L+1> – 0.36|H→L+2></td></tr>
<tr><td></td><td></td><td>2.088</td><td>593.7</td><td>0.4031</td><td>0.42|H→L+2> + 0.23|H-1 → L+1> – 0.18|H-2 → L></td></tr>
<tr><td></td><td></td><td>4.044</td><td>306.6</td><td>0.2014</td><td>0.18|H-2→L+8> + 0.20|H-5→L+2> – 0.22|H-35→L>
– 0.20|H-19→L+3></td></tr>
<tr><td>2oo(b)</td><td>I</td><td>1.720</td><td>720.8</td><td>0.0531</td><td>0.55|H-1→L+1> – 0.36|H→L+2></td></tr>
<tr><td></td><td></td><td>1.934</td><td>641.0</td><td>0.2535</td><td>0.39|H-2→L> – 0.46|H-13→L+3></td></tr>
<tr><td></td><td></td><td>1.992</td><td>622.3</td><td>0.3732</td><td>0.28|H-2→L> + 0.40|H-13→L+3></td></tr>
<tr><td></td><td></td><td>4.013</td><td>309.0</td><td>0.2911</td><td>0.34|H-2→L+8> + 0.33|H-35→L></td></tr>
<tr><td></td><td></td><td></td><td></td><td></td><td></td></tr>
<tr><td>2oc</td><td>II</td><td>1.537</td><td>806.9</td><td>0.7112</td><td>0.58|H→L> – 0.23|H-5→L></td></tr>
<tr><td></td><td></td><td>1.868</td><td>663.7</td><td>0.0926</td><td>0.38|H-2→L> – 0.27|H-5→L></td></tr>
<tr><td></td><td></td><td>3.925</td><td>315.9</td><td>0.0983</td><td>0.39|H-17→L> + 0.22|H-26→L> +0.31|H-26→L+2></td></tr>
<tr><td>2oc</td><td>I</td><td>1.402</td><td>884.1</td><td>0.6461</td><td>0.77|H → L></td></tr>
<tr><td></td><td></td><td>1.982</td><td>625.6</td><td>0.1468</td><td>0.22|H-2→L+2> + 0.32|H-14→L+4> – 0.29|H-4→L></td></tr>
</table>

		4.007	309.4	0.1657	0.30|H-2→L+8> – 0.26|H-1→L+2> – 0.22|H→L+8> + 0.32|H-34→L+1>
**2cc**	**II**	1.438	862.3	1.3931	0.51|H→L> + 0.30|H-1→L+1>
		1.837	675.1	0.1866	0.23|H→L+4> + 0.20|H-4→L> – 0.23|H-2→L+1> + 0.18|H-1→L>
		3.107	399.0	0.1077	0.28|H-1→L+1> + 0.21|H→L+4> – 0.21|H-4→L+1> – 0.18|H-4→L>
		3.369	368.0	0.1701	0.29|H-17→L> – 0.16|H-18→L> + 0.15|H-8→L>
		3.519	352.4	0.1073	0.27|H-19→L+1> + 0.19|H-19→L> – 0.18|H-9→L+1> – 0.11|H-2→L+1>
		3.917	316.5	0.1402	0.27|H-16→L+1> + 0.21|H-16→L>– 0.19|H-13→L> + 0.26|H-5→L>
**2cc**	**I**	1.247	994.3	1.4394	0.49|H→L> – 0.30|H-1→L+1>
		1.967	630.3	0.1263	0.27|H-4→L> + 0.21|H-1→L+2>
		3.533	351.0	0.2121	0.22|H-2→L+1> + 0.21|H-13→L+1>
		3.937	314.9	0.0845	0.29|H-23→L+1> – 0.20|H-10→L>– 0.15|H-5→L>
		3.989	310.8	0.0864	0.26|H-4→L+1> + 0.22|H-3→L>
**Emission**					
**2oo**	**II**	1.444	858.7	0.0170	0.65|H-4→L> – 0.15|H-2→L>
		1.851	669.7	0.1938	0.47|H-1→L+2> – 0.51|H-1→L+1> – 0.39|H-14→L+2>+ 0.27|H-12→L+2> + 0.21|H-14→L+1>
		2.074	597.8	0.1588	0.52|H→L+2> + 0.40|H-9→L+4>
		3.966	312.6	0.2401	0.33|H-2→L+8> + 0.14|H-2 → L+6> – 0.36|H-34→L>
**2oc**	**II**	1.353	916.5	0.7071	0.57|H → L> – 0.24|H-4 → L>
		1.874	661.6	0.0774	0.32|H-5→ L> + 0.29|H→L+1> – 0.43|H-2→L> – 0.25|H→H-19>
		3.806	325.8	0.0748	0.31|H-15→L> + 0.23|H-4 → L+4> – 0.25|H-5→L+5>

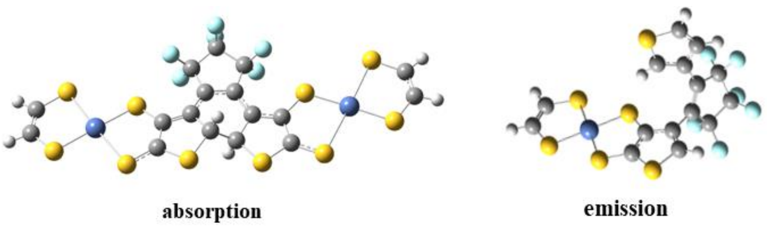

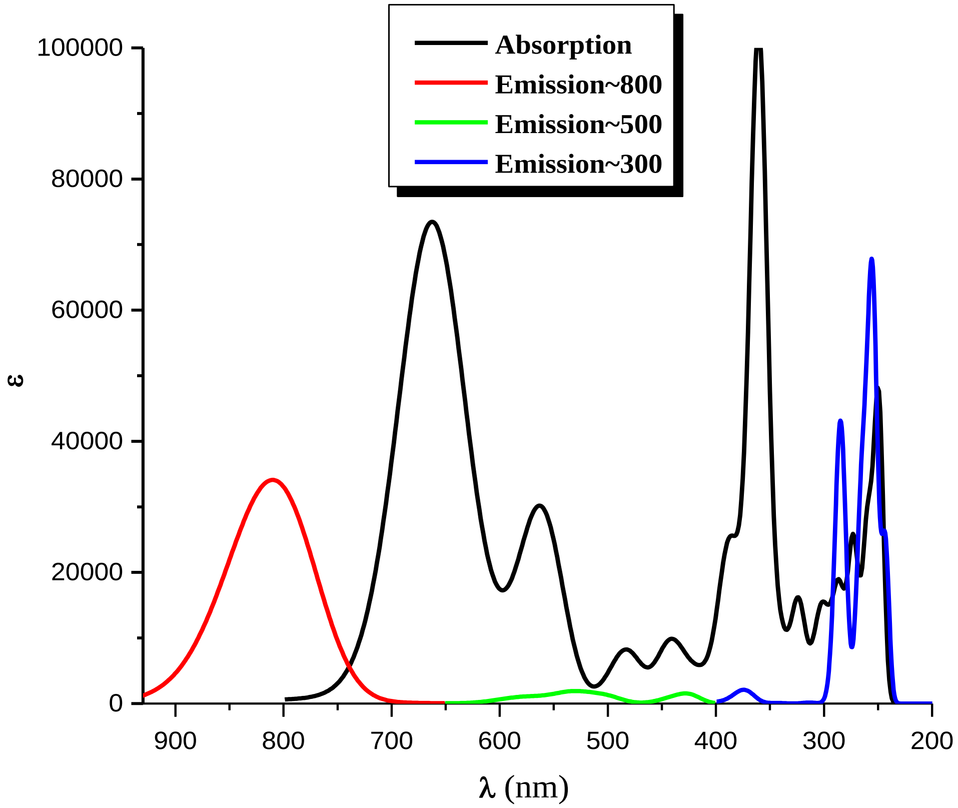

Figure S4. The absorption (black line) and the emission spectra (red, green, blue lines) of the **2oc** fragments (left fragment: Donor, right fragment: Acceptor). The CAMB3LYP/6-31G* method was used.

Absorption Spectra

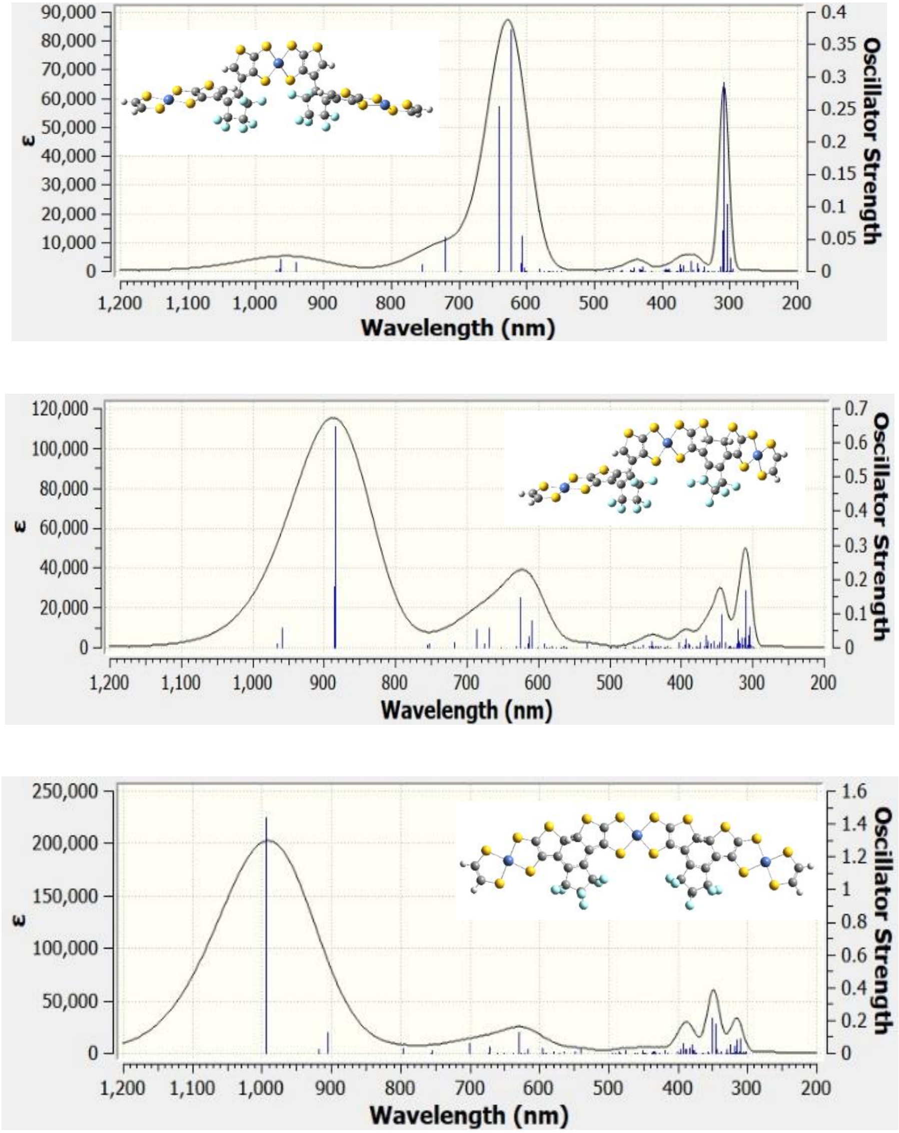

Figure S5. Absorption spectra of the **2oo**, **2oc**, and **2cc** structures at B3LYP/6-31G*(H,C,O,F,S) ECP28MWB(SDD)(Ni) level of theory. (Peak half-width at half height: 0.1 eV)

Absorption Spectra

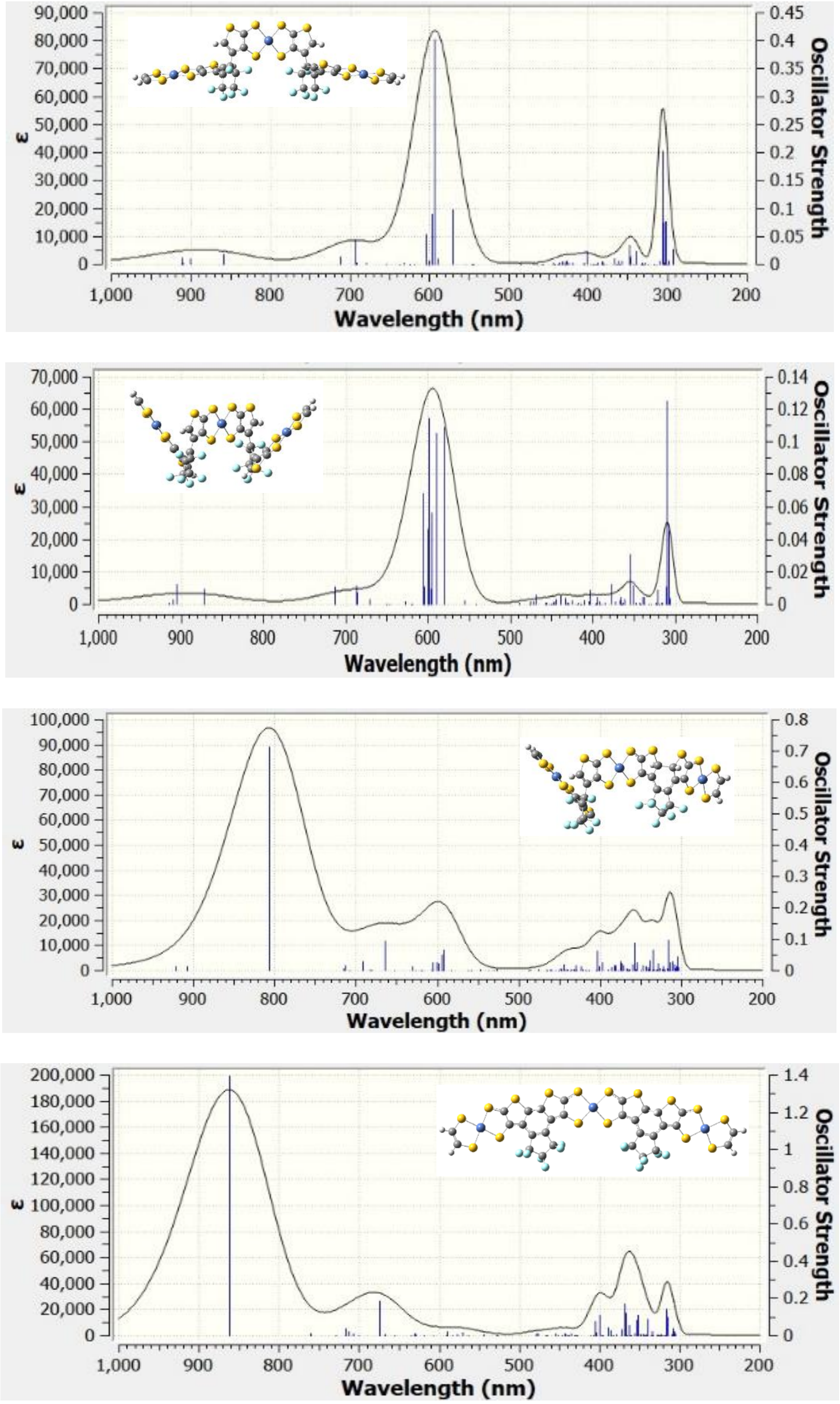

Figure S6. Absorption spectra of the **2oo**, **2oc**, and **2cc** structures at CAM-B3LYP/6-31G*H,C,O,F,S ECP28MWB(SDD)Ni level of theory. (Peak half-width at half height: 0.1 eV)

References

[1] I. D. Petsalakis, D. Tzeli, I. S. K. Kerkines and G. Theodorakopoulos, Theoretical study on the electronic structure and the absorption spectra of complexes of C60 and C59N with π-extended derivatives of tetrathiafulvalene, Comp. Theor. Chem. 965, 168-175 (2011)

[2] D. Tzeli, I. Petsalakis, G. Theodorakopoulos, Computational Insight into the Electronic Structure and Absorption Spectra of Lithium Complexes of N-confused Tetraphenylporphyrin, J. Phys. Chem. A 115, 11749-11760 (2011)
